# 

**Published:** 1859-09

**Authors:** 


ORIGINAL COMMUNICATIONS. PAGE.

ART I.—An Essay on the Treatment of Spina Bifida (*Hydrorachitis*), by Injections of Iodine. By Daniel Brainard, M.D., Prof. of Surgery in Rush Medical College, Surgeon of the Chicago City Hospital, etc. ... 517
ART. II.—Report of Two Cases of Diptheria, with Remarks. (Read before the Chicago Academy of Medical Sciences.) By DeLaskie Miller, M.D., Prof. of Obstetrics in Rush Medical College ... 539
ART. III.—A Case of Spontaneous Gangrene of Right Lower Extremity. Reported by George Schloetzer, M.D., of Chicago ... 542
ART. IV.—The Tr. Muriate Ferri in Nasal Polypus. By J. H. Reeder, M.D., of Lacon, Ill. ... 547

BOOK AND PAMPHLET NOTICES.

Report on Elephantiasis of the Scrotum, made to the Society of Surgery. By M. B[on]. H[te]. Larrey, Surgeon in Ordinary to his Majesty the Emperor. Paris. 1856 ... 548
Elements of Medicine. By Samuel Dickson, M.D. Philadelphia: Lea & Blanchard. 1859 ... 559

CORRESPONDENCE.

Communication from W. S. Robertson, M.D., Columbus City, Iowa ... 560

EXTRACTS.

On Macula Cachectica. By Dr. James Whitehead. (Third Report of the Clinical Hospital, Manchester.) ... 564
On the Treatment of Purpura Hæmorrhagica. By Dr. S. L. Hardy, Physician to the Hospital for Diseases of Children, Dublin (Dublin Hospital Gazette.) ... 564
Displacement of the Coccyx Sideways. By Dr. Ræser. (Prager Vierteljahrschr, Nov. 18th, 1858, and Edinburgh Medical Journal, Nov., 1858) ... 566

CURIOSITIES OF MEDICAL LITERATURE.

On the Delivery of the Child by Turning, as a General Rule in Labor. By Mr. E. Garland Figg, of Borrowstowness. (Medical Times and Gazette, Ranking's Abstract.) ... 568
A case of Cesarean Operation performed with success. By Borie. (Memoires de l' Academie Imperiale de Medicine. Paris, 1857-8.) ... 570
On the amount of utility of Permanent Exutories in the treatment of Chronic Diseases. By M. Zuskowski ... 570

EDITORIAL.

Opinions of the Profession concerning the "Reform School." ... 572
Chicago City Hospital ... 574
The Book Notices, Selections, etc ... 575
The Whitney Case; with Prof. Bennett's Letter ... 575
Rush Medical College.—Prof. Allen ... 580

